# Mapping perceived sentiments in university campuses with varied landscape metrics and climatic conditions

**DOI:** 10.3389/fpsyg.2024.1467966

**Published:** 2024-10-25

**Authors:** Qiyuan Yan, Yuxiang Sun

**Affiliations:** ^1^School of Education, Johns Hopkins University, Baltimore, MD, United States; ^2^Ice and Snow Tourism Resorts Equipment and Intelligent Service Technology Ministry of Culture and Tourism Key Laboratory, Jilin University, Changchun, China

**Keywords:** sustainable campus, higher education, perceived sentiment, mental health and well-being, landscape, green and blue spaces

## Abstract

A sustainable university campus should accommodate students to experience positive emotions, which can be evoked by sustainable landscape with green and blue spaces (GBS). This effect is location-dependent because local vegetative type is climate-determinative, but evidence is not sufficient for sentiments of people experiencing campus landscapes. Forty-seven university campuses were selected along a latitudinal gradient in mainland China, and 100 subjects were chosen per campus (50 indoor and 50 outdoor). Photos of the subjects’ faces on Sina Weibo were collected. Facial expressions were assigned happy, sad, and neutral scores (*n* = 4,334). The average temperature (AveT) and blue space area (BlueA) showed negative relationships with latitude, thereby generating neutral emotion scores for subjects at indoor and outdoor locations. The ratio of green space area to host campus was the only landscape metric that depressed the presentation of happiness and enhanced sadness levels. Large water bodies should be built on campuses to induce calmness, and a high ratio of green spaces should be avoided. Mapping results show that campuses in eastern regions (Beijing and Liaoning) tend to elicit positive sentiments more frequently.

## 1 Introduction

The university campus is not only a type of urban infrastructure providing higher education, but also a model of a smart city. Students, teachers, and working staffs are major users of a campus, and students account for the greatest population living in it. Their emotional perceptions in campus rule the overall educational quality and further matter for sustainable development of the host university. A campus is usually characterized as a collection of negative sentiments due to concentrated stressors from examination and graduation (Guan et al., 2017; Zhou et al., 2019). It is a critical requirement to build a sustainable campus with sustainable environment that benefits restorative effects and activates optimistic moods. Exposed to nature in green and blue spaces (GBS) can induce people perceive beneficial emotions (Scopelliti et al., 2016), which can be explained by stress reduction theory (SRT) (Ulrich et al., 1991) and attention recovery theory (ART) (Kaplan and Kaplan, 1989). Experiences with neighborhood green space can elicit perceived well-being for students in a campus (Brizhak et al., 2020; Liu et al., 2022; Wei et al., 2020a). Frequent interactions with blue space in a campus (creek, pond, or wetland) can also promote positive sentiments of students (Aghabozorgi et al., 2024; Krasny and Delia, 2014). Nature in GBS of a campus landscape accounts for the restorative effects with a variation in healing rates (Lau and Yang, 2009), students’ perceptions (Saksa and Barton, 2011), learning skills (Salih et al., 2023), and waste recycling (Abubakar et al., 2016). Current understanding about this effect was mainly derived from studies in a single campus or several at a regional scale. Limits exist in the neglect of statistical power from a higher number of places at larger scales, which further bring about more driving factors and the first two are combined landscape metrics and microclimate.

As a part of urban institutions, campus landscape has been identified to induce visitors’ sentiments with neighborhood infrastructures (Wei et al., 2020a). In urban landscape, GBS belong to a type of ecological infrastructures that play the role to benefit mental health and well-being (Ghermandi et al., 2022; Pouso et al., 2021). This effect can persist to be available in large geographical ranges in a state or up to national or cross-nation scales (Vegaraju and Amiri, 2024; Wei et al., 2022a; White et al., 2021). These pave the theoretical base to detect campus sentiments in response to spatial arrangement of GBS in a large geographical scale. In a campus, building and impervious surface are inevitably constructed and their surface feature and spatial configuration generated complex interactions with GBS that together interrupted emotional perceptions (Kistemann et al., 2023; Wu and Ren, 2021). To our knowledge, quite rare have been revealed to confirm human perceptions in built environment of campuses characterized as landscape metrics in any large scale.

Microclimates varied in different regions of GBS can be perceived as triggers of different emotions as responses (Li H. Y. et al., 2022a; Mao et al., 2022). Combined effects of regional climate and GBS landscape metrics together shaped sentiments in a wide geographical range from a local stand to a nationwide realm (Wang X. et al., 2023). University campuses can be distributed in a geographical range up to any scales, in which microclimate is an inevitable factor influencing students’ sentiments (Mallen et al., 2020). Therefore, efforts have been made to optimize the microclimatic environment in a campus to benefit emotional perceptions (Mallen et al., 2020; Sun et al., 2022). Environmental comfort is one of the key issues that campus users can strongly perceive and chase in both indoor and outdoor experiences (Konis et al., 2020; Pritoni et al., 2017). The mitigate of GBS-building landscape is a tough project, which needs a specifically designed survey to figure out across a number of campuses.

Human sentiments and perceptions are mainly assessed using self-reported scores in interviews or surveys, which are highly subjective and seldom produce 100% accurate results (Guan et al., 2021; Wei et al., 2021a). As an alternative instrument, facial data obtained from social media have been accepted as a novel source of big data by accumulating population (Sun et al., 2023; Xin et al., 2024; Zheng et al., 2023). It is easy to pool a set of big data from facial photos collected from social network services (SNSs) and used for analyzing emotional perceptions. These photos could be a source of facial expression scores that could be used to recognize the emotions of people who are exposed to the nature of a campus (Wei et al., 2020a). Therefore, the use of social media data to generate facial expression scores is suitable as a means of gauging sentiments on campuses on a large scale.

In China, university campuses are located across a large area in different climatic zones. Local GBS changes along topographic gradients have been identified, but there have been few studies in this context on campuses. The rare relevant evidence is usually in the form of theoretical explanations. In this study, we conducted a nationwide investigation with a research objective to detect the emotional perceptions of people on GBS in university campuses and potential driving forces that may be from combined landscape metrics and regional climates. The research question was whether people showed positive emotions in campuses with larger areas of GBS and what facets in landscape metrics and microclimate generated jointly contributions. To answer these questions, facial expression scores were taken as the source of emotional data which were dependent variables. Independent variables were taken as landscape metrics which can be evaluated using remote sensing technique.

## 2 Literature review

### 2.1 Facial expression scores in GBS

Facial expressions were suggested to be a type of variable in academic studies as early as 1990s, when it was commenced with studies on medicinal psychology (Haviland et al., 1996) and cultural psychology (Schimmack, 1996). Machine-based recognition broadened its use in a wider range involving food appetite since 2010s (Forestell and Mennella, 2012). It was officially introduced in studies on human emotions in GBS since 2019 (Wei et al., 2019). Currently, this technology has been used for detecting emotions toward experiences with GBS in multiple senses at local (Wei et al., 2021a; Xin et al., 2024), regional (Wang and Zhou, 2024; Wei et al., 2021b; Zheng et al., 2023), and national scales (Wang X. et al., 2023; Wei et al., 2022a; Wei et al., 2022b). Most of these studies, however, were conducted in urban parks and most of them verified ART and SRT and demonstrated benefits on perceived emotions by touching the nature in GBS. In recent chronicle, studies started to concern perceived emotions in GBS of other types of urban infrastructures, such as communities (Chen and Guo, 2022) and industrial parks (Sun et al., 2023). These results were different from those found in parks as exposure to GBS lost expected effects or even resulted in negative effects.

Otherwise, scientists always underlined the importance of GBS in campus and their benefits for emotions of students since 2010 (Bowler et al., 2010; Krasny and Delia, 2014). Recently, it was summarized in a review and novel instruments were appealed for new studies (Aghabozorgi et al., 2024). To our knowledge, only one study employed facial expression scores as a gauge to evaluate emotions of campus visitors, but results only explained a little about effects of GBS (Wei et al., 2020a). Overall, it is needed to document more trials to figure out specific effects of GBS exposure on emotions in campus.

### 2.2 Emotional perceptions toward landscape metrics of GBS

By means of remote sensing technology, it has achieved to detect the relationship between landscape metrics and human emotions in different locations of GBS. The mostly employed parameters were distance from an objective (such as the downtown of a city) (Wei et al., 2019) and GBS area (Li H. Y. et al., 2022b; Zhang et al., 2021). Digital elevation map (DEM) was frequently used to extract elevation information about GBS locations (He et al., 2023b; Liu et al., 2021a; Zhang et al., 2021). Digital surface map (DSM) was introduced to be used on basis on DEM, and their subtraction resulted in the calculation of vegetative height of GBS (He et al., 2023b; Li H. Y. et al., 2022b; Zhang et al., 2021). In detail, studies on human emotions in blue space were mainly conducted in urban wetland parks (Li H. Y. et al., 2022a; Li Y. J. et al., 2022) and waterbody in built-up regions of a city (Chen and Guo, 2022; Wei et al., 2022a). Only at regional or national scales, can a study model the relationship between coordinate of GBS and local people’s emotions (Liu et al., 2021b; Wei et al., 2022a; Wei et al., 2022b). Overall findings indicated benefits on human emotions in parks with large areas of GBS (Guan et al., 2021; Liu et al., 2021a), but this effect was neutral without sufficient number of pavements (Huang et al., 2022). In communities and industrial parks, GBS area showed no effect on perceived emotions (Chen and Guo, 2022; Sun et al., 2023). Elevation usually showed a depressing effect on perceived emotions (Li H. Y. et al., 2022b; Zhang and Wang, 2012), and vegetative height was found to be only perceived as a positive emotion trigger in degraded forest parks (He et al., 2023b). Even so, it is scarce about landscape metrics of GBS in campus especially in a large geographical scale.

### 2.3 Microclimate and emotional perception of GBS visitors

Temperature was the most concerned meteorological factor that can impose effects on GBS visitors (Li H. Y. et al., 2022a; Mao et al., 2022). Regarding that vegetations can modify microclimate, the cooling temperature was perceived as a trigger of positive sentiments in wetland parks (Li H. Y. et al., 2022a) and temperate forest parks (Mao et al., 2022). In spring, the cooling temperature in a forest park was found to activate physiological well-being of lowered diastolic blood pressure for university students (An et al., 2019). Air humidity was not fond of by GBS visitors as it was reported to cause negative emotions (Li H. Y. et al., 2022a; Wei et al., 2020b). The quiet voice in a peaceful atmosphere can evoke positive sentiments (Goodenough et al., 2024; Wei et al., 2020b). In wetland parks, wind velocity was perceived as a commencement of sad mood (Li Y. J. et al., 2022), but it was perceived as a positive effect in forest parks at highland (He et al., 2023b). Again, quite little can be referred to about microclimate in campus and human emotions.

## 3 Materials and methods

### 3.1 Study area and sampling plots

**Figure 1:**
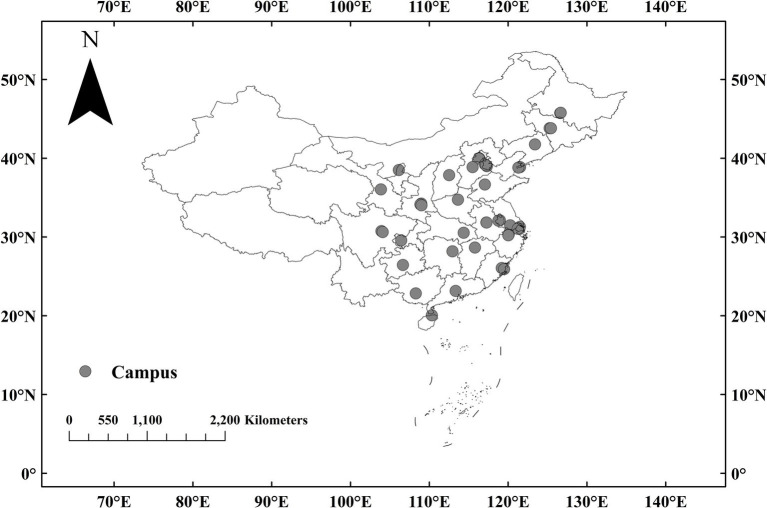
Distribution of university campuses for data curation in mainland China.

Forty university campuses were chosen in provincial or municipality regions of mainland China as plots to be investigated (Figure 1). Table 1 shows detailed information about these universities. Chosen campuses include universities with a range of disciplines (e.g., Tsinghua University, Fuzhou University, and Hainan University) and those providing professional education in a specific domain (e.g., Xi’an Jiaotong University and Tianjin Foreign Studying University). The inclusion rules for universities selected for screening are as follows:

1. Located in a province or a municipality with no more than two campuses, thereby extending the plotting area.
2. Facial photos of at least 100 subjects can be obtained from a selected campus to make sure the number of participants is sufficient for further analysis.

**Table 1:** Names and coordinates of 40 universities located at locations across mainland China.

Number	Province	City	University name	Longitude (°)	Latitude (°)
1	Anhui	Hefei	Anhui Univ.^1^	117.25	31.85
2			Univ. Sci.^2^ Tech.^3^ China	117.26	31.84
3	Beijing		Tsinghua Univ.	116.32	40.00
4			Peking Univ.	116.31	39.99
5			Beihang Univ.	116.34	39.98
6			Beijing Lang.^4^ Cult.^5^ Univ.	116.34	39.99
7			Beijing Inst.^6^ Tech.	116.31	39.96
8	Fujian	Fuzhou	Fuzhou Univ.	119.19	26.06
9			Fuzhou Univ. Int. Stud.^7^ Trade	119.49	25.93
10	Gansu	Lanzhou	Lanzhou Univ.	103.86	36.05
11	Guangdong	Guangzhou	South China Univ. Tech.	113.34	23.16
12	Guangxi	Nanning	Guangxi Univ.	108.28	22.84
13	Guizhou	Guiyang	Guizhou Univ.	106.66	26.45
14	Hainan	Haikou	Hainan Univ.	110.32	20.06
15	Hebei	Baoding	North China Electr.^8^ Power Univ.	115.50	38.88
16	Heilongjiang	Harbin	Harbin Inst. Tech.	126.63	45.74
17			Harbin Eng.^9^ Univ.	126.68	45.77
18	Hunan	Changsha	Hunan Univ.	112.94	28.18
19	Jilin	Changchun	Jilin Univ.	125.27	43.82
20			Jilin Int. Stud. Univ.	125.44	43.82
21	Jiangsu	Nanjing	Nanjing Univ.	118.95	32.12
22	Jiangsu	Wuxi	Jiangnan Univ.	120.27	31.49
23	Jiangsu	Nanjing	Southeast Univ.	118.77	32.08
24	Liaoning	Shenyang	Northeast Univ.	123.41	41.76
25	Liaoning	Dalian	Dalian Univ. Tech.	121.52	38.88
26			Dalian Univ. Foreign Lang.	121.30	38.81
27	Shaanxi	Xi’an	Xi’an Jiaotong Univ.	108.98	34.25
28			Xi’an Int. Stud. Univ.	108.87	34.14
29			Xi’an Fanyi Univ.	109.01	34.03
30	Shanghai		East China Norm.^10^ Univ.	121.40	31.23
31			Shnaghai Jiao Tong Univ.	121.43	31.03
32			Tongji Univ.	121.50	31.29
33			Shnaghai Int. Stud. Univ.	121.22	31.05
34	Sichuan	Chengdu	Univ. Electr. Sci. Tech. China	103.93	30.75
35	Tianjin		Hebei Univ. Tech.	117.05	39.24
36			Tianjin Univ.	117.31	39.00
37			Tianjin Foreign Stud. Univ.	117.20	39.11
38	Zhejiang	Hangzhou	Zhejiang Univ.	120.08	30.30
39			Zhejiang Int. Stud. Univ.	120.03	30.22
40	Chongqing		Sichuan Int. Stud. Univ.	106.43	29.57

^1Univ., university; 2Sci., science; 3Tech., technology; 4Lang., language; 5Cult., culture; 6Inst., institute; 7Stud., study; 8Electr., electricity; 9Eng., engineering; 10Norm., normal.^

Among subjects secured in rule (2), half of the subjects’ photos appear to be taken indoors and the other half outdoors.

### 3.2 Collection of facial photos and facial expression scores

Posted facial photos from social media in 2022 and 2023 were taken as the data source for rating emotional scores. As in previous studies, Sina Weibo was the chosen SNS platform. In this study, Sina Weibo was used as a platform to post advertisement that recruited volunteers to send their facial photos to a given email address. A 100 photos were required from volunteers per campus, and if more were obtained, only 100 were randomly retained. The background of a photo was visually evaluated and referred to as indoors or outdoors. Only when results were confirmed was the selected photo sent for further analysis to ensure minimum human error in visual evaluation. The two methods were also responsible for cropping and rotating the photos to ensure the highest accuracy for facial emotion recognition (Huang et al., 2022). A photo was chosen by passing a basic requirement of resolution was not lower than 50 dpi. FireFACE ver. 1.0 software was used as an instrument to recognize facial emotions and rate their scores, as used in several previous studies (Li Y. J. et al., 2022; Wei et al., 2022a; Wei et al., 2022b). Emotions were scored as happy, sad, and neutral as in earlier studies. These three emotions were identified to obtain significant matching accuracies (Guan et al., 2021). The net positive emotion performance, namely the positive response index (PRI), was calculated using the happy score minus the sad score. Finally, a total of 4,334 photos were successfully recognized for their facial emotions and related to emotional scores.

### 3.3 Remote evaluation of landscape metrics

ArcGIS (ver. 10.2, Esri Branch, Shanghai, China) was used to outline every campus in a computer–human interaction process according to dots in Baidu Maps (Baidu, 2023) in a method adapted from Zhang et al. (2021). Campus area (CampusA) was evaluated by the software using the sum of the pixels framed in the outlined area. The elevation of every campus was established using data from the digital elevation model (DEM) (NASA EarthData, 2023). Vegetation height (VegH) was assessed using the difference between data assessed from the digital surface model (DeWitt et al., 2015) and the DEM in areas of pixels occupied by green spaces. Areas of GBS on were evaluated using the extent of assembled pixels in green and blue colors, respectively. The green space area was evaluated using the normalized difference vegetation index (NDVI) (Chen and Guo, 2022):


(1) $$\mathrm{NDVI}=\frac{{\mathrm{Band}}_{5}-{\mathrm{Band}}_{4}}{{\mathrm{Band}}_{5}+{\mathrm{Band}}_{4}}$$

where, Band_4_ is the red light band, and Band_5_ is the near-infrared light band. The blue space area (BlueA) was evaluated using a modified normalized difference water index (MNDWI) (Sekertekin et al., 2018):


(2) $$\mathrm{MNDWI}=\frac{{\mathrm{Band}}_{3}-{\mathrm{Band}}_{1}}{{\mathrm{Band}}_{3}+{\mathrm{Band}}_{1}}$$

where, Band_1_ is the shortwave infrared light band, and Band_3_ is the green light band. Green space area ratio (GreenR) and blue space area ratio (BlueR) were calculated as areas of GBS divided by that of the particular campus (CampusA), respectively.

### 3.4 Regional meteorological factors and air quality

Meteorological conditions on the day a photo was posted were described by the meteorological factors of highest temperature (MaxT), lowest temperature (MinT), and rainfall (Rain) from the nearest meteorological station of the network of Climate Data Center of the National Meteorological Information Center of China (National Meteorological Information Centre, 2024). AveT was calculated as the mean between MaxT and MinT. Although it has been shown that velocity rate may also have partial effects on people’s emotional perceptions (He et al., 2023b), data about wind flow in this study were mostly recorded as zero, and these data were not included. The air quality index (AQI) was found to impact the expression of negative sentiments (Wang X. et al., 2023). The AQI data were also documented for analysis from neighboring stations in the China General Environmental Monitoring Network (China National Environmental Monitoring Centre, 2023).

### 3.5 Data process and analysis

Data were analyzed and processed using SAS software version 9.4 (SAS Inst., Cary, NC, USA). Data distributions were diagnosed using histogram bar charts, which can be referred to as patterns for detecting normal distribution on the basis of the Shapiro–Wilk test. Facial expressions were divided into indoor, outdoor, and pooled indoor and outdoor. All data that failed to follow the normal distribution patterns were ranked and used for analyses in general linear models, and raw data were transferred back when results were disclosed. Collinearity among abiotic factors of landscape metrics, climatic factors, and air quality was tested, and factors that had a variance inflation factor (VIF) higher than 10.0 were excluded from further analysis (Chen et al., 2023). Analysis of variance was used to detect the effects of combined location variation (degree of freedom [*df*] = 39), photo location (*df* = 1), year of photographing (*df* = 1), and season when photos were taken (*df* = 3). When significant effects were detected, the results were arranged and compared using the Duncan test. Parameters for landscape metrics, climatic factors, and air quality were plotted along latitudinal gradients, fitted by linear curves. The structural equation model was used to detect inner relationships across the latent variables of geography, climate, and landscape and their contributions to facial expression scores. Multivariate linear regression was employed to detect the combined effects of landscape metrics and climatic factors on facial expression scores.

## 4 Results

### 4.1 Data characteristics

**Figure 2:**
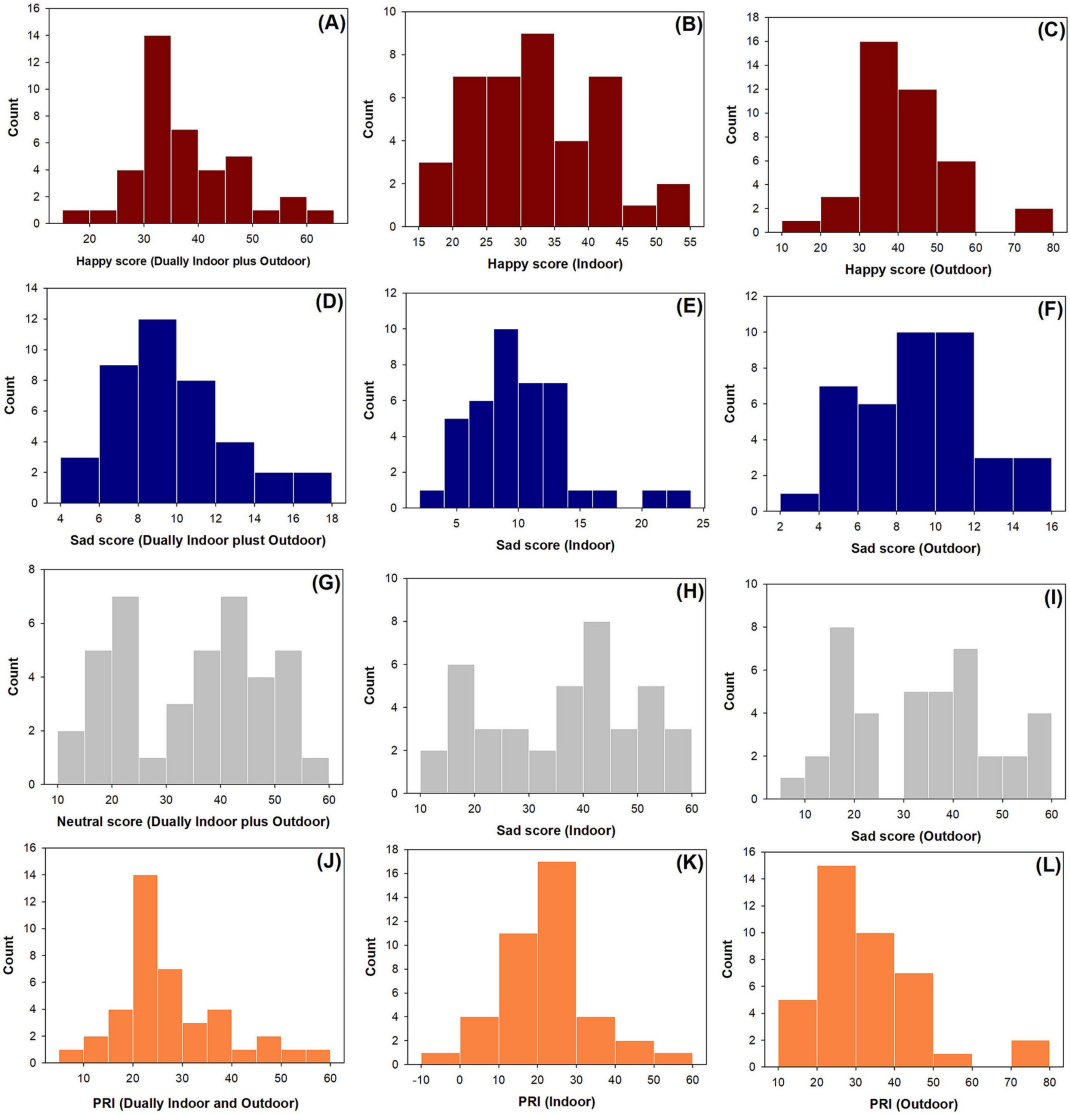
Histograms of counts for happy (A–C), sad (D–F), neutral (G–I), and PRI (J–L) scores split in pools of dually indoor and outdoor data (A,D,G,J), indoor data (B,E,H,K), and outdoor data (C,F,I,L).

Happy emotion scores ranged from 17.55 to 62.00%, 15.73–54.22%, and 18.58–79.65% with averages of 37.49% ± 8.97% (mean ± standard deviation), 32.55% ± 9.16, and 42.41% ± 12.57% for pooled indoor and outdoor data (Figure 2A), indoor data (Figure 2B), and outdoor data (Figure 2C). According to the Shapiro–Wilk test, happy scores followed the normal distributions for data dually indoor and outdoor (*p* = 0.0974) (Figure 2A) and indoor (*p* = 0.7399) (Figure 2B), but data on happy scores for outdoor data did not follow the normal distribution (*p* = 0.0157) (Figure 2C). The Cronbach alpha was 0.71 for data of happy scores.

Sad emotion scores ranged from 5.21 to 16.62%, 3.65–23.05%, and 2.64–15.98% with averages of 9.67% ± 2.93, 10.12% ± 4.03, and 9.21% ± 3.07% for pooled dually indoor and outdoor data (Figure 2D), indoor data (Figure 2E), and outdoor data (Figure 2F). Sad scores followed normal distributions for data dually indoor and outdoor (*p* = 0.0651) and outdoor data (*p* = 0.6961), but sad scores for the indoor data did not follow the normal distribution (*p* = 0.0039). The Cronbach alpha was 0.78 for data of happy scores.

Neutral emotion scores ranged from 11.70 to 57.98%, 11.35–59.60%, and 9.73–56.71% with averages of 34.89% ± 13.06, 36.34% ± 13.62, and 33.46% ± 14.08% for pooled dually indoor and outdoor data (Figure 2G), indoor data (Figure 2H), and outdoor data (Figure 2I). Neutral scores followed normal distributions for data dually indoor and outdoor (*p* = 0.0594), indoor data (*p* = 0.0541), and outdoor data (*p* = 0.0529).

PRI emotion scores ranged from 9.35 to 55.56%, −7.32 to 50.56%, and 10.32–76.70% with averages of 27.85% ± 10.06, 22.43% ± 11.10, and 33.24% ± 14.21% for pooled dually indoor and outdoor data (Figure 2J), indoor data (Figure 2K), and outdoor data (Figure 2L). PRI scores followed normal distributions for indoor data (*p* = 0.8305), but PRI scores did not follow the normal distributions for indoor data (*p* = 0.0324) and outdoor data (*p* = 0.0054).

### 4.2 Changes in atmospheric factors along latitude gradient

Collinearity was tested for all facial expression scores as dependent variables, and results revealed that only the independent variables of MaxT, MinT, and AveT had VI*F* values higher than 10.0. Therefore, AveT was left on behalf of all temperature parameters.

**Figure 3:**
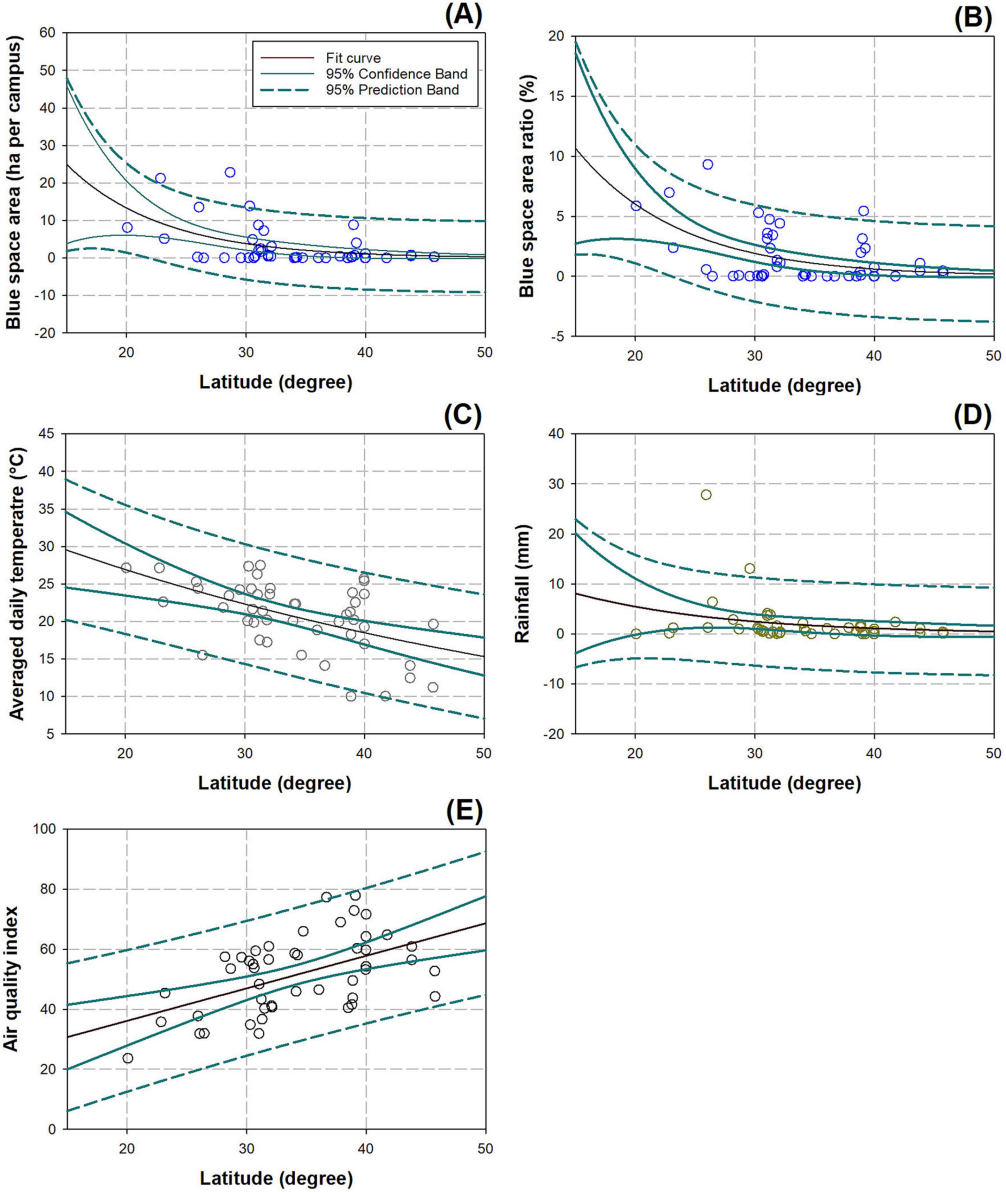
Correlations between latitudes of campus locations and abiotic factors in blue space area (A), blue space area ratio (B), average daily temperature (C), rainfall (D), and AQI (E). Curve fits are tinted in red color with 95% confidence band and 95% prediction band disclosed as well.

The relationship between latitude of campus location and BlueA was negative, which can be fitted by an exponential decay model (Figure 3A):


(3) $$\mathrm{BlueA}=176.2326\times e^{-0.1324\times Lat\text{.}}$$

where, *R*^2^ = 0.2878 and *p* = 0.0009. The relationship between latitude and BlueR was negative, which can also be fitted by an exponential decay model (Figure 3B):


(4) $$\mathrm{BlueR}=41.1981\times e^{-0.0972\times Lat\text{.}}$$

where, *R*^2^ = 0.2524 and *p* = 0.0011. The relationship between latitude and AveT was negative and fitted by an exponential decay model (Figure 3C):


(5) $$\mathrm{AveT}=35.5642\times e^{-0.0181\times Lat\text{.}}$$

where, *R*^2^ = 0.2820 and *p* < 0.0011. Rain showed a negative relationship with latitude, which can be fitted by an exponential decay model (Figure 3D):


(6) $$\mathrm{Rain}=798.0554\times e^{-0.1871\times Lat\text{.}}$$

where, *R*^2^ = 0.3610 and *p* = 0.0002. Finally, AQI had a positive relationship with latitude, which can be fitted by a linear curve (Figure 3E):


(7) AQI=1.1150×Lat.+12.0079

where, *R*^2^ = 0.3334 and *p* < 0.0001.

### 4.3 Combined effects of time and location on facial expression scores

The difference in data across the years from 2022 to 2023 had no effects on facial expression scores. Therefore, yearly data were pooled. Most scores showed significant differences among the four seasons, or at different locations (indoor vs. outdoor) for different campuses.

**Figure 4:**
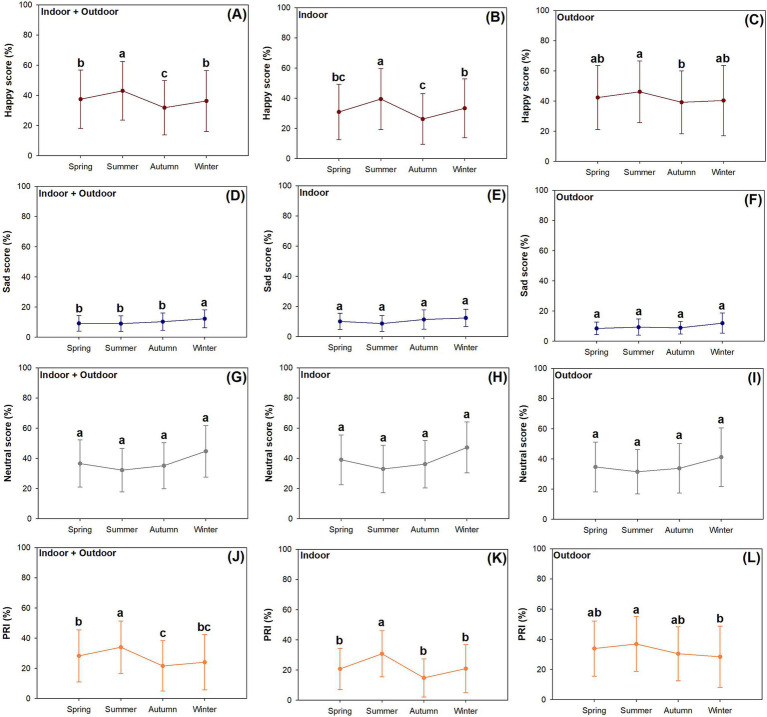
Seasonal variations of happy (A–C), sad (D–F), neutral (G–I), and PRI (J–L) emotion scores extracted from photos taken at places dually indoor and outdoor (A,D,G,J), solely indoor (B,E,H,K), and solely outdoor (C,F,I,L). Different letters mark significant differences (ranked if necessary) among seasons according to repeated measured results in ANOVA using Duncan test at 0.05 level. Dots mark means of raw data and error bars present standard errors.

Over the four seasons, happy scores increased in summer and decreased in autumn compared to spring, which rose back to an unchanged level in winter (*F* = 5.56; *p* < 0.0001) (Figure 4A). Happy scores for indoor data also showed higher levels in summer than in spring, and they were higher in winter than in summer (*F* = 3.09; *p* < 0.0001) (Figure 4B). Happy scores for outdoor data did not show any significant difference in spring and in the other three seasons, but values in autumn were lower than in summer (*F* = 6.99; *p* = 0.0001) (Figure 4C).

Sad scores showed an increasing trend among the four seasons and were higher in winter than in earlier seasons for data collected from both indoor and outdoor places (*F* = 4.35; *p* = 0.0045) (Figure 4D). Both indoor (*F* = 2.33; *p* = 0.0725) and outdoor data (*F* = 0.78; *p* = 0.5074) on sad scores showed no responses to seasonal variation (Figures 4E,F).

Neutral scores were not significantly responsive to seasonal variation for data collected from both indoor and outdoor places (*F* = 1.65; *p* = 0.1766) (Figure 4G), indoor data (*F* = 0.67; *p* = 0.5682) (Figure 4H), and outdoor winter data (*F* = 0.28; *p* = 0.8387) (Figure 4I).

Data on PRI scores collected from both indoor and outdoor places showed the following fluctuations: when compared to the value in spring, there was an increase in summer and a decline in autumn (*F* = 18.25; *p* < 0.0001) (Figure 4J). Indoor data on PRI scores were highest in summer among all four seasons (*F* = 9.52; *p* < 0.0001) (Figure 4K). Outdoor data of PRI scores were lower in winter than in summer (*F* = 5.82; *p* = 0.0006) (Figure 4L).

**Figure 5:**
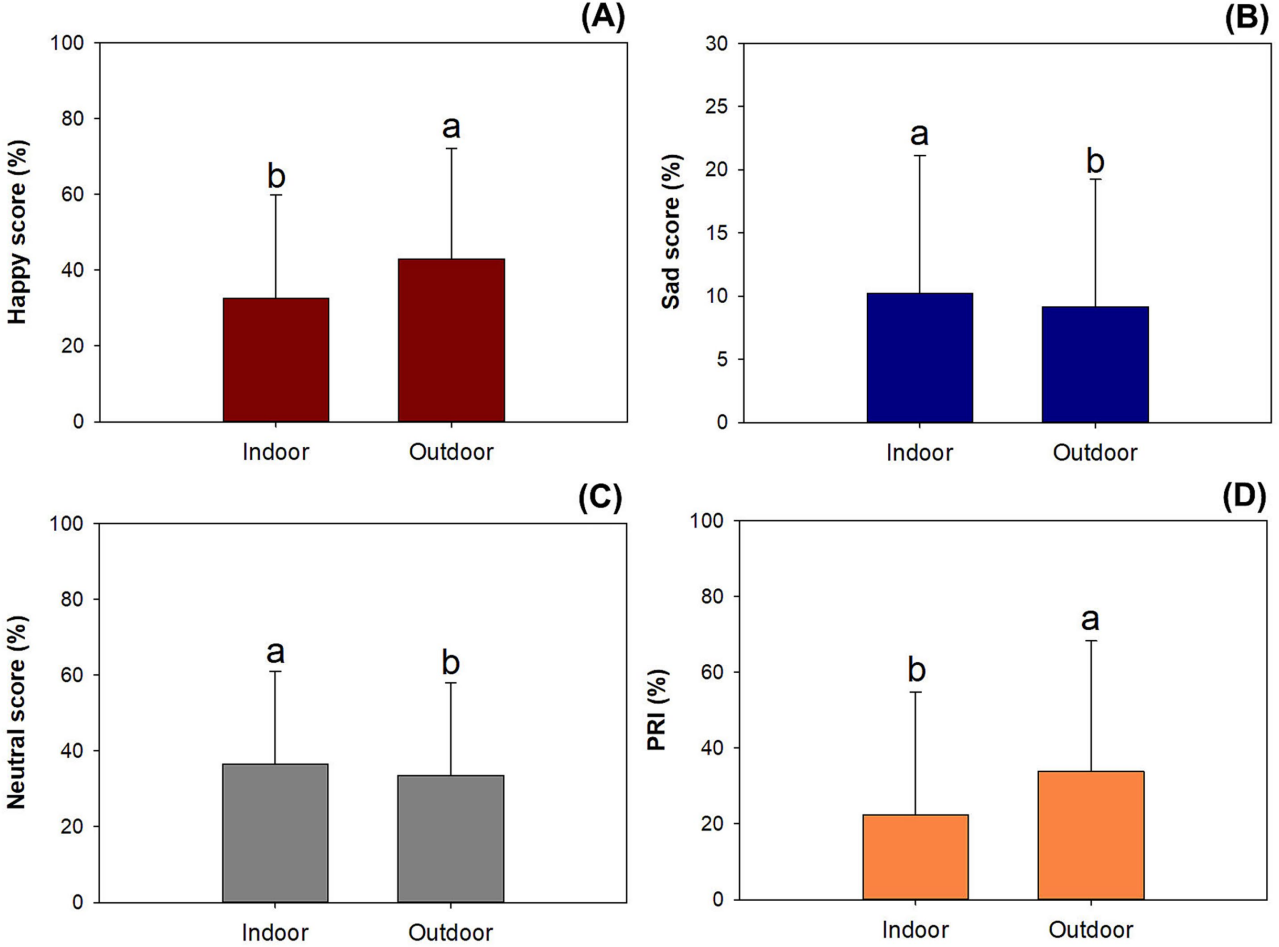
Facial expression scores collected at indoor and outdoor locations in university campuses for happy (A), sad (B), neutral (C), and PRI scores (D). Different letters indicate significant difference according to Duncan test at 0.05 level. Error bars present standard errors.

Compared to emotional scores for indoor data, the outdoor data resulted in higher happy scores (*F* = 74.37; *p* < 0.0001) (Figure 5A). In contrast, outdoor data was lower for sad (*F* = 5.62; *p* = 0.0178) (Figure 5B) and neutral scores (*F* = 8.94; *p* = 0.0028) (Figure 5C) compared to indoor data. Again, outdoor data increased the PRI relative to indoor data (*F* = 64.72; *p* < 0.0001) (Figure 5D).

**Figure 6:**
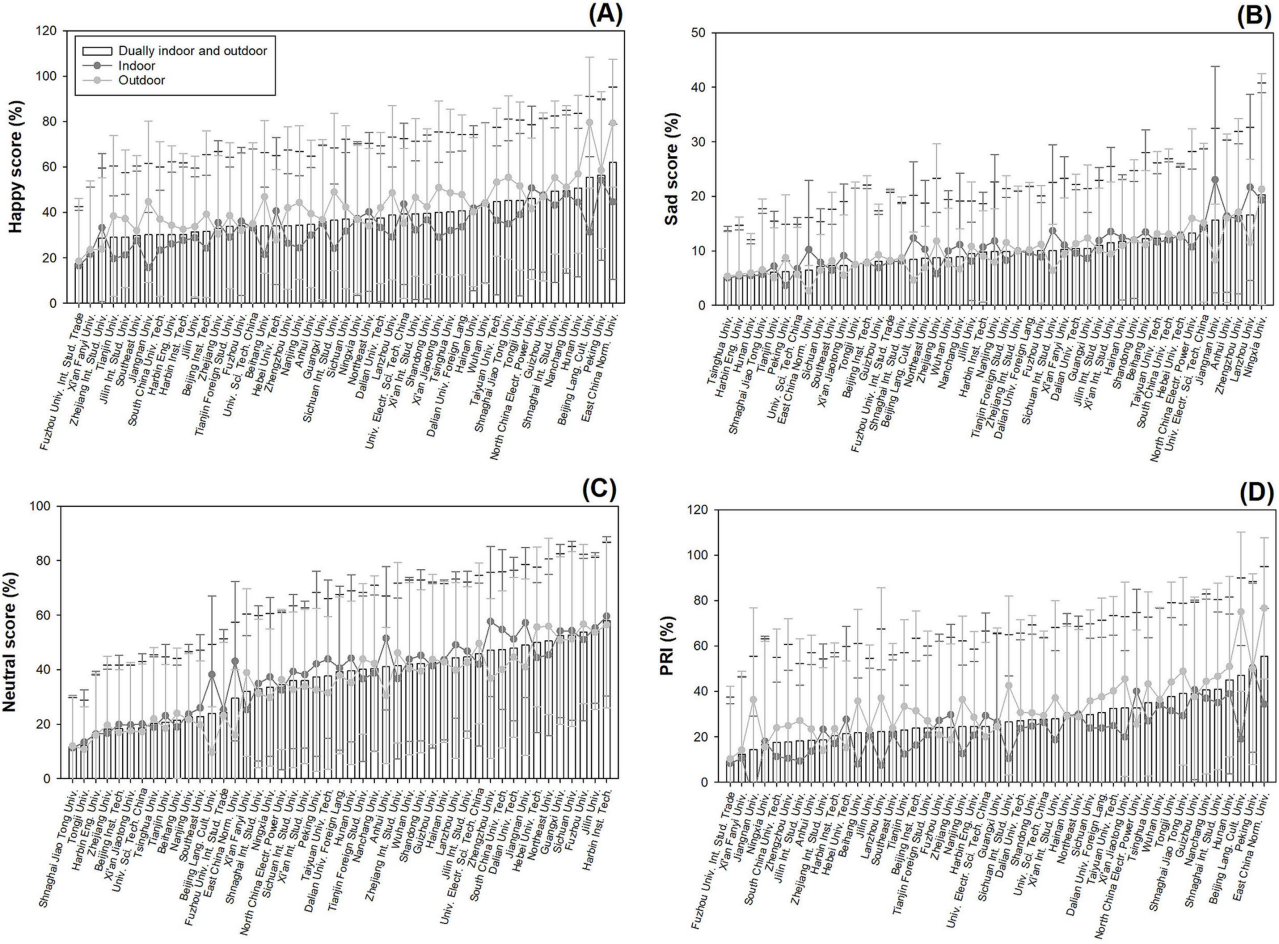
Ranks of facial expression scores for happy (A), sad (B), neutral (C), and PRI (D) scores assessed using data collected at dually indoor and outdoor places, solely indoor places, and solely outdoor places following increasing orders among university campuses. Columns present averages of data collected from dually indoor and outdoor places. Dots in deep gray color indicate data collected from indoor places and dots in light gray color indicate data from outdoor places. Error bars mark standard errors.

The variation among university campuses resulted in significant responses for happy (*F* = 5.56; *p* < 0.0001) (Figure 6A), sad (*F* = 3.97; *p* < 0.0001) (Figure 6B), neutral (*F* = 9.35; *p* < 0.0001) (Figure 6C), and PRI (*F* = 4.94; *p* < 0.0001) (Figure 6D) using data collected dually at indoor and outdoor places. Indoor data on happy scores (*F* = 3.09; *p* < 0.0001) were mostly lower than outdoor data (*F* = 5.11; *p* < 0.0001) (Figure 6A).

For sad scores, however, indoor data (*F* = 3.67; *p* < 0.0001) were mostly close to outdoor data (*F* = 2.31; *p* < 0.0001) (Figure 6B). Occasionally, campuses of East China Normal University, Beijing Language Culture University, Sichuan International Studying University, and Jiangnan University showed higher sad scores for indoor data compared to outdoor data.

Neutral scores showed higher levels for indoor data (*F* = 8.51; *p* < 0.0001) than outdoor data (*F* = 9.35; *p* < 0.0001) in campuses of the Beijing Language Culture University, East China Normal University, Anhui University, South China University of Technology, and Jiangnan University (Figure 6C).

PRI scores were generally higher for outdoor data (*F* = 4.61; *p* < 0.0001) compared to indoor data (*F* = 3.26; *p* < 0.0001) (Figure 6D).

### 4.4 Structural equation models

**Figure 7:**
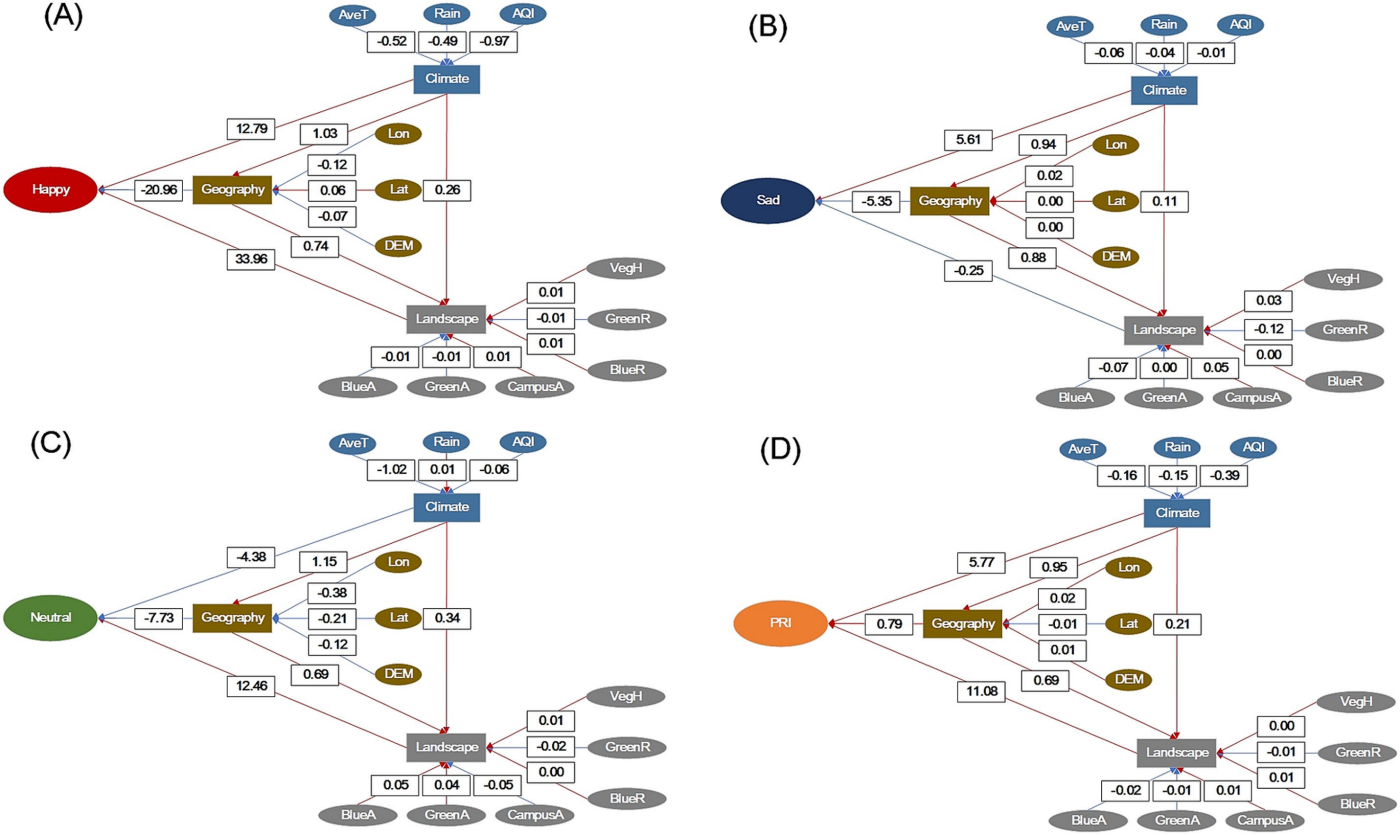
Structural equation models assessing facial expression scores of happy (A), sad (B), neutral (C), and PRI scores (D) against latent variables of climate, geography, and landscape for university campuses in China. Numerics in black-color lined outlines indicate eigenvalues of the path effect. Red-color arrows indicate positive path effects and blue-color arrows indicate negative path effects.

The *p* value in model Chi-square for happy score was lower than 0.0001 with goodness of fit index (GFI) of 0.9613, adjusted GFI (AGFI) of 0.4198, parsimonious GFI (PGFI) of 0.0739, and normed fit index (NFI) of 0.9862. The latent variables of climate and landscape had positive path effects on happy scores, while geography had a negative effect (Figure 7A). For climate, all three fixed factors of AveT, Rain, and AQI had negative path effects; for geography, longitude, and elevation had negative path effects while latitude had a positive path effect; for landscape, GBS areas showed negative path effects as did GreenR, while CampusA, BlueR, and VegH showed positive path effects.

The *p* value in model Chi-square for sad score was lower than 0.0001 with goodness of fit index (GFI) of 0.9713, AGFI of 0.5647, PGFI of 0.0747, and NFI of 0.9942. The latent variable of climate had a positive path effect on sad scores, but geography and landscape both showed negative path effects (Figure 7B). Again, the three fixed factors showed negative path effects on climate. In contrast, the factors all showed positive path effects on geography. GBS areas and GreenR showed negative path effects on landscape, while other factors showed positive effects.

The *p* value in model Chi-square for neutral score was lower than 0.0001 with goodness of fit index (GFI) of 0.9196, AGFI of −0.2195, PGFI of 0.0707, and NFI of 0.9372. The latent variables of climate and geography showed negative path effects on neutral scores, while landscape had a positive path effect (Figure 7C). Rain showed a positive path effect on climate, while AveT and AQI showed negative effects. All three factors showed negative path effects on geography. For landscape, CampusA and GreenR showed negative effects, and GBS areas, VegH, and BlueR showed positive effects.

The *p* value in model Chi-square for neutral score was lower than 0.0001 with goodness of fit index (GFI) of 0.9696, AGFI of 5,395, PGFI of 0.0746, and NFI of 0.9928. The latent variables of climate, geography, and landscape all showed positive path effects on PRI. The three fixed factors showed negative path effects on climate; geography, longitude, and elevation showed positive path effects, while latitude showed a negative path effect. The GBS areas and GreenR showed negative path effects on landscape, while other fixed factors showed positive effects.

### 4.5 Multivariate linear regression

GreenR showed negative parameter effects on happy scores for data collected from both indoor and outdoor places and solely outdoor places (Table 2). For sad scores, longitude showed negative effects on data from both indoor and outdoor places and on data from solely indoor places. In addition, GreenR had a positive effect on sad scores for both indoor and outdoor places. AveT showed negative effects on neutral scores in both indoor and outdoor data. Latitude showed negative effects on neutral scores for indoor–outdoor data. BlueA had positive effects on the neutral scores of all three types, and CampusA had a negative effect on the indoor data of neutral scores. Only GreenR showed negative effects on the PRI using indoor–outdoor data and solely outdoor data.

**Table 2:** Parameter estimates of multivariate linear regression for happy, sad, and neutral emotions of visitors standing at indoor and outdoor locations against landscape, geographical, and climatic factors in university campuses across mainland China.

Latent variable	Fixed factor	Parameter	*F* value	*p* value	Parameter	*F* value	*p* value	Parameter	*F* value	*p* value
		Happy-Both^1^			Happy-Indoor			Happy-Outdoor		
–	Intercept	41.11 ± 2.27	333.03	<0.0001				47.80 ± 3.20	224.03	<0.0001
Climate	AveT^2^									
	Rain^3^									
	AQI^4^									
Geography	Longitude									
	Latitude									
	Elevation									
Landscape	BlueA^5^									
	GreenA^6^									
	BlueR^7^									
	GreenR^8^	−0.11 ± 0.05 ^9^	4.76	0.0354				−0.15 ± 0.07	4.49	0.0408
	CampusA^10^									
	VegH^11^									
		**Sad-Both**			**Sad-Indoor**			**Sad-Outdoor**		
	Intercept	27.38 ± 8.41	10.60	0.0024	49.54 ± 13.69	13.09	0.0009			
Climate	AveT									
	Rain									
	AQI									
Geography	Longitude	−0.16 ± 0.07	5.07	0.0304	−0.29 ± 0.11	7.38	0.0101			
	Latitude									
	Elevation									
Landscape	BlueA									
	GreenA									
	BlueR									
	GreenR	0.03 ± 0.02	4.39	0.0431						
	CampusA									
	VegH									
		**Neutral-Both**			**Neutral-Indoor**			**Neutral-Outdoor**		
	Intercept	101.67 ± 15.35	43.85	<0.0001	193.26 ± 39.43	24.02	<0.0001	96.74 ± 17.59	30.25	<0.0001
Climate	AveT	−2.41 ± 0.41	35.25	<0.0001	−2.53 ± 0.43	35.17	<0.0001	−2.42 ± 0.47	26.95	<0.0001
	Rain									
	AQI									
Geography	Longitude									
	Latitude	−0.93 ± 0.32	8.52	0.0060	−0.76 ± 0.29	6.99	0.0122	−0.83 ± 0.37	5.21	0.0284
	Elevation									
Landscape	BlueA	1.08 ± 0.39	7.64	0.0090	1.88 ± 0.48	15.37	0.0004	1.17 ± 0.45	6.85	0.0129
	GreenA									
	BlueR									
	GreenR									
	CampusA				−0.06 ± 0.02	6.15	0.0181			
	VegH									
		**PRI-Both**			**PRI-Indoor**			**PRI-Outdoor**		
	Intercept	32.97 ± 2.49	175.10	<0.0001				39.64 ± 3.59	122.09	<0.0001
Climate	AveT									
	Rain									
	AQI									
Geography	Longitude									
	Latitude									
	Elevation									
Landscape	BlueA									
	GreenA									
	BlueR									
	GreenR	−0.14 ± 0.06	6.67	0.0138				−0.18 ± 0.08	5.01	0.0311
	CampusA									
	VegH									

^1Both, emotional scores collected from subjects standing both at indoor locations or outdoor; 2AveT, average temperature; 3Rain, rainfall; 4AQI, air quality index; 5BlueA, blue space area; 6GreenA, green space area; 7BlueR, blue space area ratio to campus area; 8GreenR, green space area ratio to campus area; 9values are means ± standard errors; 10CampusA, campus area; 11VegH, vegetation height.^

### 4.6 Mapping sentiments synthesizing landscape and climatic factors

**Figure 8:**
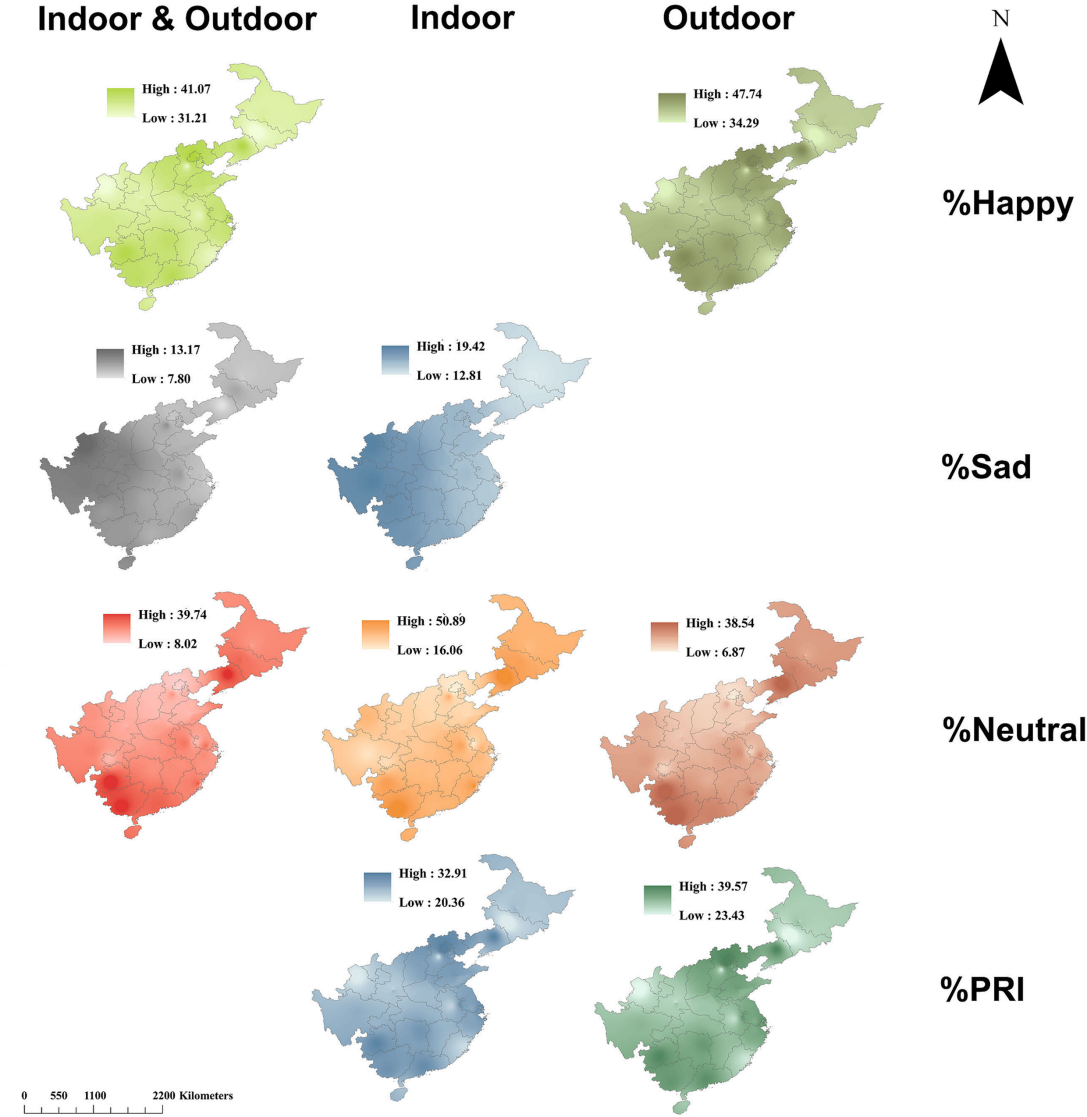
Mapping interpolations of sentiments in university campuses for indoor and outdoor visitors using data across layers with coefficients adapted from Table 2.

**Figure 9:**
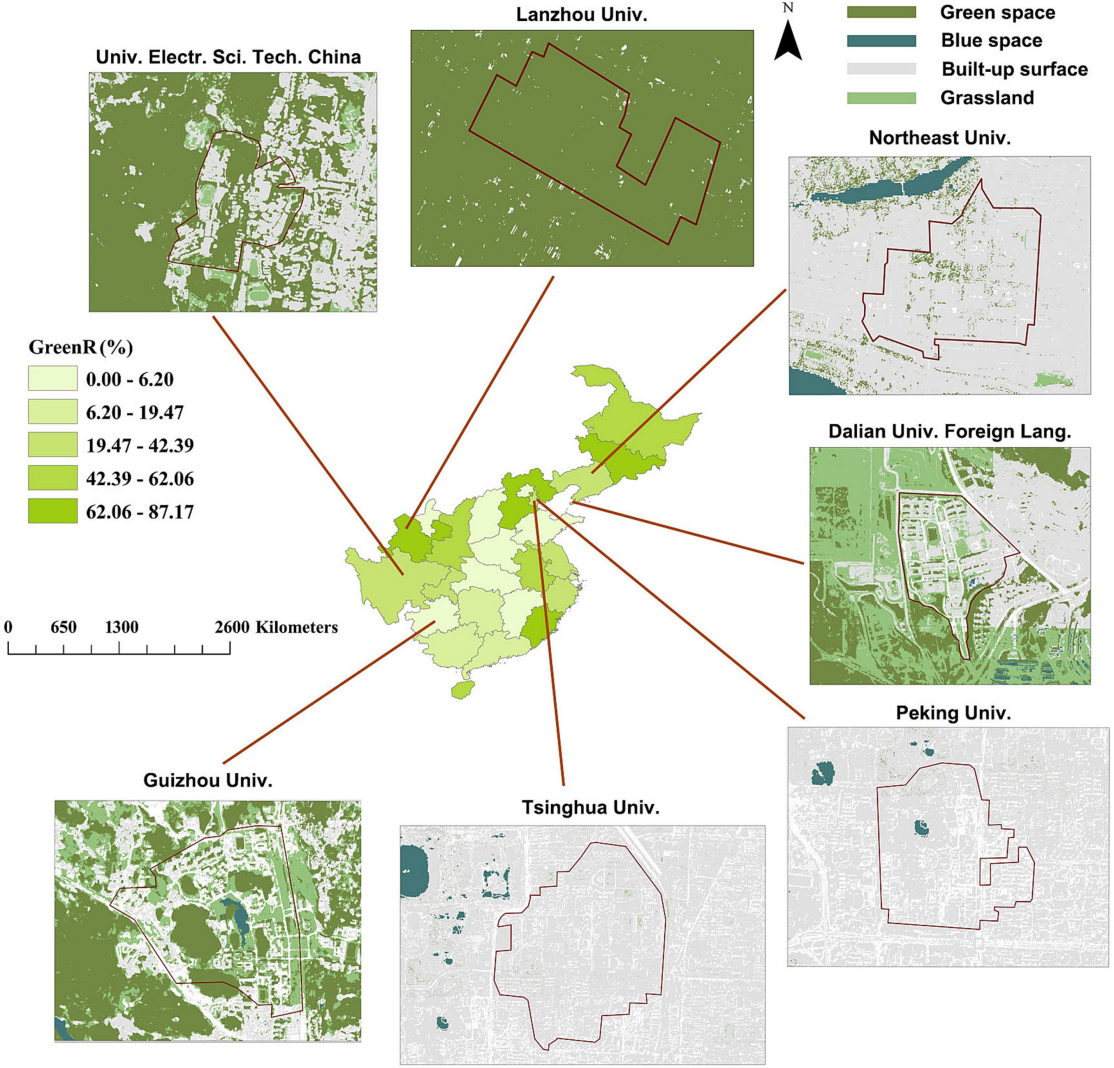
Spatial distribution of provincial areas colored by magnitude of area ratio of green space to host campus (GreenR) dominated by typical campuses chosen for this study. Area of campus is outlined by full lines in color of dark red.

By synthesizing the regressions in Table 2, the spatial distributions of sentiments can be mapped against landscape metrics and microclimates (Figure 8). Happy scores showed high levels distributed in campuses located in two regions of East China and Southwest China. The northern aggregation distribution of high happiness was indicated in areas across north Hebei, Beijing, and Liaoning, and the southern aggregation was indicated in Guizhou and Guangxi (Figure 9). Sad scores showed a general decreasing trend with the increase in longitude. Neutral scores showed a similar distribution pattern to that of happy scores with higher levels existing in campuses located in Hebei, Anhui, and Fujian.

## 5 Discussion

### 5.1 Contrasting photographing locations: indoor vs. outdoor

On a campus, time spent by visitors at indoor places is of a higher frequency than outdoor time. Indoor people showed lower positive emotions but higher sadness than those took photos outdoor. In a campus, most photos were exposed by students, and they spent more attention for academic work at indoor places than at outdoor locations. According to ART, the strong consumption of attention can result in reduction of mental well-being which further caused more perceptions of bad moods. The small area of a campus results in a high frequency of people encountering alternatively indoor or outdoor occasions (Chen et al., 2018). Outdoor visitors’ facial expressions can be largely driven by combined perceptions about landscape and atmosphere (He et al., 2023a; Wang X. et al., 2023). Emotions of the indoor population may also be influenced by outdoor conditions, such as air pollution (Mohammadyan et al., 2017). Green space in the ecological infrastructure of a campus can be seen through windows by indoor students (van den Bogerd et al., 2020). Enjoyment of outdoor landscapes can fuel energy for indoor activities (Tochaiwat et al., 2023). In this study, outdoor people generally showed higher scores for positive sentiments than inside people, thereby concurring with a previous judgment that people look happier when they are outside (Bowler et al., 2010). This results from a higher frequency of exposure to GBS in outdoor places than in indoor places. The indoor environment was likelier to be perceived as a stressful condition, especially in campuses with more social science classes. Students in this type of university usually take more examinations. Therefore, they were more inclined to be impacted by the indoor environment than those in universities with science and technology domains.

### 5.2 Time-dependent effect on facial expression scores

The uses of happy and sad scores as sources of data had been validated in previous studies (Guan et al., 2021; Wei et al., 2021a). It was reported that analyses of happy and sad scores had higher matching accuracies than recognitions of other types of facial emotions. The fact was that most of other emotion scores (e.g., contempt or angry) failed to pass the significant value of matching accuracy. In addition, the Cronbach alpha ratio was higher than 0.70 in our study. This was identified to be an acceptable range of reliability in practice using facial expression scores as a source of data (Kuhlmann and Margraf, 2023).

Year for data collection included pandemic and recovery years, but no yearly difference was found, and the pandemic did not affect the emotional perceptions of people on campuses. However, university students were reported to have perceived strong stress and loneliness during the COVID-19 lockdown in Germany (Werner et al., 2021). University workers were also found to have perceived anxiety and depression during the pandemic in Spain (Salazar et al., 2021). These negative emotions were perceived during the pandemic, but the associated research results are not comparable to ordinary times.

Positive sentiments showed seasonal variations, but negative emotion scores showed unclear dynamics. This can be explained by the formation of facial expressions, which reflect happiness in time-dependent frequency (Diener et al., 2009). Users of SNS platforms are more likely to show happiness than sadness in posted photos. Summer is the season when most Chinese universities fall in episodes at the end of a semester or high education, when students are more likely to feel happy and share their joyful perceptions on SNS. This can be explained by SRT as people can perceive more positive moods when they were experiencing an episodic when mental stress was reduced. New semesters usually start in autumn when more freshmen begin their university education, and they mostly look neither happy nor sad at the time of enrollment.

### 5.3 Driving forces of abiotic factors in shaping facial expression scores

GreenR was the only landscape metric that contributed to the decline of happiness score and the increase of sadness score mainly for outdoor populations. Although GreenA showed similar path effects to GreenR, the area effect failed to impose any significant effects on happy scores. The general goodness of fit in SEM was acceptable for happy scores due to the high GFI of 0.9613 (Stone, 2021). These findings differed from the current understanding that large areas of urban greening precondition positive public sentiments. Current studies were mainly conducted in urban parks, that attract leisure-seeking visitors and random passers-by mainly with leisure or relaxing aims. In a campus where most users are students, competitive senses and concerns about academic performance limited their perceptions of positive moods as they did in parks. It was reported that university students can perceive declines in perceptions of examination stress and relationship state when touching the nature in green space, but their worries were still unreleased even strengthened for concerns about post-graduate uncertainties and performance evaluated by teachers (Guan et al., 2017; Zhou et al., 2019). In a campus, green space may have been taken as a ‘fence’ that hinder students walking to a building to study, which evoked negative emotions. A large area of green space could encourage more people to experience greenery on a campus, but a large area of campus would reduce the frequency of experiencing green nature (Sun et al., 2023). That is why GreenR is more predictable than GreenA for eliciting positive sentiments.

BlueA had a negative relationship with latitude. However, it was also found to be positively correlated with latitude in urban parks in eastern cities of China (Wei et al., 2022a). These results increase uncertainties regarding whether the relevant waterbody has a negative or a positive effect on human emotion. According to Li H. Y. et al. (2022a), people’s emotions were elicited by touching water partly due to perceptions of regional climate. However, in our study, AveT was perceived as a driver of neutral emotions, suggesting people’s neutral emotions toward temperature. The effect of microclimate on neutral scores cannot be fully reliable due to negative value of AGFI. This was caused by a relatively large number of observations (*n* = 40) with only a small Chi-square degree of freedom (6.0) (Schermellech-Engel and Moosbrugger, 2003). Therefore, the neutral emotion was mainly ruled by different perceptions about areas of blue spaces distributed along the latitude gradient.

### 5.4 Mapping spatial distributions of campus sentiments

We used social media data and spatial interpolation to map the spatial distributions of the sentiments of people on campuses in China who posted facial photos online. Our study was endorsed by a mechanism through which human sentiments can be predicted in a region where all the abiotic factors were arranged to bring about the theoretical driving effects of perceived emotions. We used this methodology to map sentiments against landscape and microclimate on campuses in different places. Similar interpolations have been employed successfully for urban park visitors against combined microclimate and air pollution (He et al., 2023a; Wang X. et al., 2023) and landscape metrics in green spaces (Zhang et al., 2021) and blue spaces (Li H. Y. et al., 2022b; Li Y. J. et al., 2022). Therefore, the approach is reliable for mapping sentiments on campuses. Our mapping results indicate that people on campuses in Beijing and Liaoning showed higher positive sentiments than in other regions, in line with the findings of a previous study (Wei et al., 2020a). Campuses in Southwestern China were also found to benefit from positive sentiments. Campuses in these regions all had a high GreenR, which accounted for the mapping results.

### 5.5 Theoretical implication

Our study findings have implication meanings. In theory, our study verified theories about exposure to GBS and emotional responses in university campuses, but our results disagreed to those findings in previous trials conducted in parks (Schrammeijer et al., 2021; Scopelliti et al., 2016). In brief, the area ratio of green space contributed to perceptions of negative emotions and blue space area frequently attracted people with neutral emotions. These findings enriched current understandings about ART and SRT by repudiating all-good responses in emotional perceptions (Bowler et al., 2010). This difference was generated because subjects were mostly with aimless visits in parks, but in campus most people have stressful affairs. Exposure to nature means reduction in attention and evokes stressful perceptions that together elicit negative emotions and control perceptions of positive sentiments. Similar results were found in industrial parks where most workers were also highly stressed and exposure to GBS stimulated presentation of negative moods of them (Sun et al., 2023). Overall, it can be concluded that positive consequences explained by ART and SRT should be preconditioned for subjects who were unlikely population being subjected to high mental stressors.

### 5.6 Managerial implication

If positive moods were purchased as a goal for students, a university campus should be planned with low ratios of GBS in touchable spaces with frequent visits. This managerial suggestion is essential because negative effects of exposure to GBS on emotional perceptions were found on more subjects exposing photos at outside places than inside buildings. This contradicts with other managerial suggestions for campus planning that encouraged high rate of frequency for GBS exposure (Saksa and Barton, 2011; Salih et al., 2023; Tochaiwat et al., 2023; Wang X. F. et al., 2023). Although microclimate was considered to be a major driver that shaped students’ moods in a campus (Mallen et al., 2020; Sun et al., 2022; van den Bogerd et al., 2020; Wang X. F. et al., 2023), the significant effects of meteorological factors vanished in our study which suggests no needs to care about microclimate’s influence. This difference may be caused by research scale variation and our study employed a national scale on which longitude showed a strong impact to counter sadness perception. This suggests a positioning rule that a happiness-induced campus should be placed in eastern coastal cities with dense populations and developed local economies (Wei et al., 2022a). To avoid perceptions of sad emotions, essential GBS can be constructed in places with rare people passing by; for example, planting trees along the inside part of a wall or building a small water pond behind the dormitory.

### 5.7 Limitations of this study

First, the background on which subjects were screened in their locations was determined visually according to the background of the photos. Two technicians sorted indoor vs. outdoor types of photos. However, we admit that technical errors cannot be completely eliminated. A more precise survey could split indoor and outdoor subjects by recruiting participants and conducting real-time investigations. Second, we did not find yearly differences in facial expression data across 2022 and 2023, but it was suspected that the pandemic lockdown in 2022 reinforced some of the errors in perception. Those who experienced forced lockdown may have perceived mental stress and anxiety. Therefore, future research could repeat our experiment in a period free from pandemic interruption. Finally, although the technique has been queried and accepted several times, facial photos collected from SNS platforms may be subject to question due to the facial expressions being posted. We agree with explanations in previous studies that have been accepted by academic referees that all photos were collected under the same situation and errors are of the same level. However, we also agree that photo sampling can be more practical by collection in off-line real senses, e.g., from camera records. Furthermore, we surmise that most people who posted their facial photos to SNS platforms were probably students. Compared to workers and resort visitors, students are more willing to share photos of their faces online. Based on earlier research experience (Guan et al., 2021), we suggest validating online facial expression scores using off-line subjects before further data is collected.

### 5.8 Future work suggestions

As a place with highly knowledge concentrated, a campus is suggested to be evaluated for people’s emotions toward experiences therein by involving the cultural context. It has been confirmed that cultural background has a solid effect on facial expression (Schimmack, 1996), and cultural variation can affect students’ senses of happiness (Brizhak et al., 2020). Future work can be upgraded to collect real-time photos of people in a campus with their cultural backgrounds categorized in advance. It can also consider to investigate facial expressions in special types of host universities, such as language-major campuses vs. technical science campuses.

## Data Availability

The datasets presented in this article are not readily available because the study is still going on and data are subjected to classified contract. Requests to access the datasets should be directed to the corresponding author YS.

## References

[ref1] Abubakar I. R. al-Shihri F. Ahmed S. M. (2016). Students’ assessment of campus sustainability at the University of Dammam, Saudi Arabia. Sustain. For. 8:59. doi: 10.3390/su8010059

[ref2] Aghabozorgi K. van der Jagt A. Bell S. Smith H. (2024). How university blue and green space affect students' mental health: a scoping review. Urban For. Urban Green. 97:128394. doi: 10.1016/j.ufug.2024.128394

[ref3] An B. Y. Wang D. Liu X. J. Guan H. M. Wei H. X. Ren Z. B. (2019). The effect of environmental factors in urban forests on blood pressure and heart rate in university students. J. For. Res. 24, 27–34. doi: 10.1080/13416979.2018.1540144

[ref4] Baidu (2023). Baidu map: to go where you want to go. Available at: https://map.baidu.com/ (Accessed January 2, 2024).

[ref5] Bowler D. E. Buyung-Ali L. M. Knight T. M. Pullin A. S. (2010). A systematic review of evidence for the added benefits to health of exposure to natural environments. BMC Public Health 10:10. doi: 10.1186/147-2458-10-456 20684754 PMC2924288

[ref6] Brizhak Z. Kolesina K. Mironenkova N. (2020). "Positive psychology in students' axiological choice situations", in: 8th annual international scientific and practical conference on innovative technologies in science and education (ITSE), 2003, 210.

[ref7] Chen L. W. Chen T. P. Chen D. E. IEEE (2018). “iGuiding: a Mobile campus care and guidance system based on internet of things technologies” in IEEE international conference on pervasive computing and communications (PerCom).

[ref8] Chen C. H. Guo P. (2022). Can indoor residents perceive green and blue spaces in communities as posted sentiments? A verification in Nanchang. Forests 13:1421. doi: 10.3390/f13091421

[ref9] Chen B. Yang Y. T. Chen L. Y. Jiang L. Hong Y. Zhu J.. (2023). Microclimate along an elevational gradient controls foliar litter cellulose and lignin degradation in a subtropical forest. Front. For. Global Change 6:1134598. doi: 10.3389/ffgc.2023.1134598

[ref10] China National Environmental Monitoring Centre (2023). China National Environmental Monitoring Centre. Available at: https://www.cnemc.cn (Accessed January 2, 2024).

[ref11] DeWitt J. D. Warner T. A. Conley J. F. (2015). Comparison of DEMS derived from USGS DLG, SRTM, a statewide photogrammetry program, ASTER GDEM and LiDAR: implications for change detection. GIScience Remote Sens. 52, 179–197. doi: 10.1080/15481603.2015.1019708

[ref12] Diener E. Sandvik E. Pavot W. (2009). “Happiness is the frequency, not the intensity, of positive versus negative affect” in Assessing well-being: the collected works of Ed Diener. ed. Diener E. (Dordrecht: Springer Netherlands), 213–231.

[ref13] Forestell C. A. Mennella J. A. (2012). More than just a pretty face. The relationship between infant's temperament, food acceptance, and mothers' perceptions of their enjoyment of food. Appetite 58, 1136–1142. doi: 10.1016/j.appet.2012.03.005, PMID: 22407135 PMC3340480

[ref14] Ghermandi A. Depietri Y. Sinclair M. (2022). In the AI of the beholder: a comparative analysis of computer vision-assisted characterizations of human-nature interactions in urban green spaces. Landsc. Urban Plan. 217:104261. doi: 10.1016/j.landurbplan.2021.104261

[ref15] Goodenough A. Urquhart J. Morrison K. Black J. E. Courtney P. Potter C. (2024). Using a socially-engaged arts approach to exploring how diverse socio-cultural groups accessed, valued, engaged with and benefited from an urban treescape during the COVID-19 pandemic. Urban For. Urban Green. 98:128398. doi: 10.1016/j.ufug.2024.128398

[ref16] Guan H. M. Wei H. X. Hauer R. J. Liu P. (2021). Facial expressions of Asian people exposed to constructed urban forests: accuracy validation and variation assessment. PLoS One 16:126699:e0253141. doi: 10.1371/journal.pone.0253141, PMID: 34138924 PMC8211262

[ref17] Guan H. Wei H. He X. Ren Z. An B. (2017). The tree-species-specific effect of forest bathing on perceived anxiety alleviation of young-adults in urban forests. Ann. For. Res. 327–341. doi: 10.15287/afr.2017.897

[ref18] Haviland J. M. WalkerAndrews A. S. Huffman L. R. Toci L. Alton K. (1996). Intermodal perception of emotional expressions by children with autism. J. Dev. Phys. Disabil. 8, 77–88. doi: 10.1007/bf02578441

[ref19] He Q. Wang Y. Qiu Q. Su Y. Wang Y. Wei H. X.. (2023a). Joint effects of air PM_2.5_ and socioeconomic dimensions on posted emotions of urban green space visitors in cities experiencing population urbanization: a pilot study on 50 cities of East China. Sci. Total Environ. 861:160607. doi: 10.1016/j.scitotenv.2022.160607, PMID: 36460101

[ref20] He Q. Wang Y. Qiu Q. Su Y. Wei H. X. Li J. Y. (2023b). Posted sentiments toward experiences in degraded forests are shaped jointly by landscape structure and microclimate. Ecosyst. Health Sustain. 9:0004. doi: 10.34133/ehs.0004

[ref21] Huang S. Y. Zhu J. J. Zhai K. B. Wang Y. Wei H. X. Xu Z. H.. (2022). Do emotional perceptions of visible greeneries rely on the largeness of green space? A verification in Nanchang, China. Forests 13:1192. doi: 10.3390/f13081192

[ref22] Kaplan R. Kaplan S. (1989). The experience of nature: a psychological perspective. New York, NY, USA: Cambridge University Press.

[ref23] Kistemann T. Zerbe S. Säumel I. Fehr R. (2023). Urban green and blue spaces in times of climate change. Gesundheitswesen 85, S296–S303. doi: 10.1055/a-2144-5404, PMID: 37972601

[ref24] Konis K. Blessenohl S. Kedia N. Rahane V. (2020). TrojanSense, a participatory sensing framework for occupant-aware management of thermal comfort in campus buildings. Build. Environ. 169:106588. doi: 10.1016/j.buildenv.2019.106588

[ref25] Krasny M. E. Delia J. (2014). Campus sustainability and natural area stewardship: student involvement in adaptive comanagement. Ecol. Soc. 19:27. doi: 10.5751/ES-06787-190327

[ref26] Kuhlmann B. Margraf J. (2023). A new short version of the facial expressions of emotion: stimuli and tests (FEEST) including prototype and morphed emotional stimuli. Front. Psychol. 14:1198386. doi: 10.3389/fpsyg.2023.1198386, PMID: 37941762 PMC10628552

[ref27] Lau S. Yang F. (2009). Introducing healing gardens into a compact university campus: design natural space to create healthy and sustainable campuses. Landsc. Res. 34, 55–81. doi: 10.1080/01426390801981720

[ref28] Li H. Y. Peng J. X. Jiao Y. Ai S. S. (2022b). Experiencing urban green and blue spaces in urban wetlands as a nature-based solution to promote positive emotions. Forests 13:473. doi: 10.3390/f13030473

[ref29] Li Y. J. Sun Y. X. Zhao Y. Wang Y. Cheng S. P. (2022). Mapping seasonal sentiments of people visiting blue spaces in urban wetlands: a pilot study on inland cities of China. Front. Ecol. Evol. 10:969538. doi: 10.3389/fevo.2022.969538

[ref30] Li H. Y. Wang X. G. Wei H. X. Xia T. T. Liu M. N. Ai S. S. (2022a). Geographical distribution and driving meteorological forces of facial expressions of visitors in urban wetland parks in eastern China. Front. Earth Sci. 10:781204. doi: 10.3389/feart.2022.781204

[ref31] Liu S. B. Ji Y. F. Li J. Peng Y. Li Z. T. Lai W. B.. (2022). Analysis of students' positive emotions around the green space in the university campus during the COVID-19 pandemic in China. Front. Public Health 10:888295. doi: 10.3389/fpubh.2022.888295, PMID: 36016888 PMC9395969

[ref32] Liu P. Liu M. N. Xia T. T. Wang Y. T. Guo P. (2021a). The relationship between landscape metrics and facial expressions in 18 urban Forest parks of northern China. Forests 12:1619. doi: 10.3390/f12121619

[ref33] Liu P. Liu M. N. Xia T. T. Wang Y. T. Wei H. X. (2021b). Can urban Forest settings evoke positive emotion? Evidence on facial expressions and detection of driving factors. Sustain. For. 13:8687. doi: 10.3390/su13168687

[ref34] Mallen E. Bakin J. Stone B. Sivakumar R. Lanza K. (2020). Thermal impacts of built and vegetated environments on local microclimates in an Urban University campus. Urban Clim. 32:100640. doi: 10.1016/j.uclim.2020.100640

[ref35] Mao B. Liang F. Li Z. Z. Zheng W. Q. (2022). Microclimates potentially shape spatial distribution of facial expressions for urban forest visitors: a regional study of 30 parks in North China. Sustain. For. 14:1648. doi: 10.3390/su14031648

[ref36] Mohammadyan M. Ghoochani M. Kloog I. Abdul-Wahab S. A. Yetilmezsoy K. Heibati B.. (2017). Assessment of indoor and outdoor particulate air pollution at an urban background site in Iran. Environ. Monit. Assess. 189:235. doi: 10.1007/s10661-017-5951-1 28451957

[ref37] NASA EarthData (2023). NASA EarthData: digital elevation model product. Available at: https://search.earthdata.nasa.gov/search/?ac=true&m=0.0703125!0!2!1!0!0%2C2 (Accessed January 2, 2024).

[ref38] National Meteorological Information Centre (2024). China Meteorological Data Service Centre. Available at: https://data.cma.cn/en (Accessed January 2, 2024).

[ref39] Pouso S. Borja A. Fleming L. E. Gómez-Baggethun E. White M. P. Uyarra M. C. (2021). Contact with blue-green spaces during the COVID-19 pandemic lockdown beneficial for mental health. Sci. Total Environ. 756:143984. doi: 10.1016/j.scitotenv.2020.143984, PMID: 33277006 PMC7688424

[ref40] Pritoni M. Salmon K. Sanguinetti A. Morejohn J. Modera M. (2017). Occupant thermal feedback for improved efficiency in university buildings. Energ. Buildings 144, 241–250. doi: 10.1016/j.enbuild.2017.03.048

[ref41] Saksa K. Barton S. S. (2011). Student perception of campus sustainable landscapes: University of Delaware Laird Campus case study. HortScience 46:S266.

[ref42] Salazar A. Palomo-Osuna J. de Sola H. Moral-Munoz J. A. Dueñas M. Failde I. (2021). Psychological impact of the lockdown due to the COVID-19 pandemic in university workers: factors related to stress, anxiety, and depression. Int. J. Environ. Res. Public Health 18:4367. doi: 10.3390/ijerph18084367 33924133 PMC8074294

[ref43] Salih S. A. Ismail S. Ujang N. Mustafa F. A. Ismail N. A. (2023). Pocket settings for enhancing social learning experience on campus ground: a verbal-visual preference survey. Ain Shams Eng. J. 14:102134. doi: 10.1016/j.asej.2023.102134

[ref44] Schermellech-Engel K. Moosbrugger H. (2003). Evaluating the fit of structural equation models: tests of significance and descriptive goodness-of-fit measures. Methods Psychol. Res. Online 8, 23–74.

[ref45] Schimmack U. (1996). Cultural influences on the recognition of emotion by facial expressions - individualistic or Caucasian cultures? J. Cross-Cult. Psychol. 27, 37–50. doi: 10.1177/0022022196271003

[ref46] Schrammeijer E. A. van Zanten B. T. Verburg P. H. (2021). Whose park? Crowdsourcing citizen's urban green space preferences to inform needs-based management decisions. Sustain. Cities Soc. 74:103249. doi: 10.1016/j.scs.2021.103249

[ref47] Scopelliti M. Carrus G. Adinolfi C. Suarez G. Colangelo G. Lafortezza R.. (2016). Staying in touch with nature and well-being in different income groups: the experience of urban parks in Bogota. Landsc. Urban Plan. 148, 139–148. doi: 10.1016/j.landurbplan.2015.11.002

[ref48] Sekertekin A. Abdikan S. Marangoz A. M. (2018). The acquisition of impervious surface area from LANDSAT 8 satellite sensor data using urban indices: a comparative analysis. Environ. Monit. Assess. 190:381. doi: 10.1007/s10661-018-6767-3, PMID: 29881995

[ref49] Stone B. M. (2021). The ethical use of fit indices in structural equation modeling: recommendations for psychologists. Front. Psychol. 12:783226. doi: 10.3389/fpsyg.2021.783226, PMID: 34887821 PMC8650002

[ref50] Sun Y. Ma X. T. Liu Y. F. Meng L. Q. (2023). Salary satisfaction of employees at workplace on a large area of planted land. Land 12:2075. doi: 10.3390/land12112075

[ref51] Sun B. Zhang H. Zhao L. Qu K. C. Liu W. H. Zhuang Z. C.. (2022). Microclimate optimization of school campus landscape based on comfort assessment. Buildings 12:1375. doi: 10.3390/buildings12091375

[ref52] Tochaiwat K. Rinchumphu D. Sundaranaga C. Pomsurin N. Chaichana C. Khuwuthyakorn P.. (2023). The potential of a tree to increase comfort hours in campus public space design. Energy Rep. 9, 184–193. doi: 10.1016/j.egyr.2023.05.258

[ref53] Ulrich R. S. Simons R. F. Losito B. D. Fiorito E. Miles M. A. Zelson M. (1991). Stress recovery during exposure to natural and urban environments. J. Environ. Psychol. 11, 201–230. doi: 10.1016/S0272-4944(05)80184-7

[ref54] van den Bogerd N. Dijkstra S. C. Koole S. L. Seidell J. C. de Vries R. Maas J. (2020). Nature in the indoor and outdoor study environment and secondary and tertiary education students' well-being, academic outcomes, and possible mediating pathways: a systematic review with recommendations for science and practice. Health Place 66:102403. doi: 10.1016/j.healthplace.2020.102403, PMID: 32932004

[ref55] Vegaraju A. Amiri S. (2024). Urban green and blue spaces and general and mental health among older adults in Washington state: analysis of BRFSS data between 2011-2019. Health Place 85:103148. doi: 10.1016/j.healthplace.2023.103148 38043153

[ref56] Wang X. F. Chen Z. Q. Ma D. W. Zhou T. T. Chen J. T. Jiang X. (2023). Relationship between visual and thermal comfort and electrodermal activity in campus blue-green spaces: a case study of Guangzhou, China. Sustainability 15:11742. doi: 10.3390/su151511742

[ref57] Wang X. Meng L. Liu Y. Wei H. (2023). Facial expressions of urban forest visitors jointly exposed to air pollution and regional climate. Forests 14:1571. doi: 10.3390/f14081571

[ref58] Wang L. Zhou C. W. (2024). Evaluation of perceptions using facial expression scores on ecological service value of blue and green spaces in 61 parks in Guizhou. Sustain. For. 16:4108. doi: 10.3390/su16104108

[ref59] Wei H. X. Hauer R. J. Chen X. He X. Y. (2019). Facial expressions of visitors in forests along the urbanization gradient: what can we learn from selfies on social networking services? Forests 10:1049. doi: 10.3390/f10121049

[ref60] Wei H. Hauer R. J. Guo S. (2021b). Daytime dynamic of spontaneous expressions of pedestrians in an urban forest park. Urban For. Urban Green. 65:127326. doi: 10.1016/j.ufug.2021.127326

[ref61] Wei H. Hauer R. J. He X. (2021a). A forest experience does not always evoke positive emotion: a pilot study on unconscious facial expressions using the face reading technology. Forest Policy Econ. 123:102365. doi: 10.1016/j.forpol.2020.102365

[ref62] Wei H. Hauer R. J. Sun Y. Meng L. Guo P. (2022a). Emotional perceptions of people exposed to green and blue spaces in forest parks of cities at rapid urbanization regions of East China. Urban For. Urban Green. 78:127772. doi: 10.1016/j.ufug.2022.127772

[ref63] Wei H. X. Hauer R. J. Zhai X. Q. (2020a). The relationship between the facial expression of people in university campus and Host-City variables. Appl. Sci. Basel 10:1474. doi: 10.3390/app10041474

[ref64] Wei H. X. Ma B. Q. Hauer R. J. Liu C. Y. Chen X. He X. Y. (2020b). Relationship between environmental factors and facial expressions of visitors during the urban forest experience. Urban For. Urban Green. 53:126699. doi: 10.1016/j.ufug.2020.126699

[ref65] Wei H. Zhang J. Xu Z. Hui T. Guo P. Sun Y. (2022b). The association between plant diversity and perceived emotions for visitors in urban forests: a pilot study across 49 parks in China. Urban For. Urban Green. 73:127613. doi: 10.1016/j.ufug.2022.127613

[ref66] Werner A. M. Tibubos A. N. Mülder L. M. Reichel J. L. Schäfer M. Heller S.. (2021). The impact of lockdown stress and loneliness during the COVID-19 pandemic on mental health among university students in Germany. Sci. Rep. 11:22637. doi: 10.1038/s41598-021-02024-5, PMID: 34811422 PMC8609027

[ref67] White M. P. Elliott L. R. Grellier J. Economou T. Bell S. Bratman G. N.. (2021). Associations between green/blue spaces and mental health across 18 countries. Sci. Rep. 11:8903. doi: 10.1038/s41598-021-87675-0, PMID: 33903601 PMC8076244

[ref68] Wu Z. F. Ren Y. (2021). The influence of greenspace characteristics and building configuration on depression in the elderly. Build. Environ. 188:107477. doi: 10.1016/j.buildenv.2020.107477

[ref69] Xin B. Zhu C. Geng J. Liu Y. (2024). Emotional perceptions of thermal comfort for people exposed to green spaces characterized using streetscapes in urban parks. Land 13:1515. doi: 10.3390/land13091515

[ref70] Zhang J. T. Wang C. H. (2012). Biodiversity and ecosystem functioning: exploring large-scale patterns in mainland China. iForest Biogeosci. Forest. 5, 230–234. doi: 10.3832/ifor0627-005

[ref71] Zhang J. Yang Z. Chen Z. Guo M. Guo P. (2021). Optimizing urban Forest landscape for better perceptions of positive emotions. Forests 12:1691. doi: 10.3390/f12121691

[ref72] Zheng Y. Zhu J. L. Wang S. Guo P. (2023). Perceived economic values of cultural ecosystem services in green and blue spaces of 98 urban wetland parks in Jiangxi, China. Forests 14:273. doi: 10.3390/f14020273

[ref73] Zhou C. W. Yan L. B. Yu L. F. Wei H. X. Guan H. M. Shang C. F.. (2019). Effect of short-term forest bathing in urban parks on perceived anxiety of young-adults: a pilot study in Guiyang, Southwest China. Chinese Geogr. Sci. 29, 139–150. doi: 10.1007/s11769-018-0987-x

